# Dichlorido[tris­(1*H*-benzimidazol-2-ylmeth­yl)amine-κ^4^ *N*,*N* ^3^,*N* ^3′^,*N* ^3′′^]iron(III) chloride tetra­hydro­furan monosolvate monohydrate

**DOI:** 10.1107/S1600536809023423

**Published:** 2009-06-27

**Authors:** Dong’e Wang

**Affiliations:** a Department of Chemistry, Kashgar Teachers College, Kashgar 844000, People’s Republic of China

## Abstract

In the title compound, [FeCl_2_(C_24_H_21_N_7_)]Cl·C_4_H_8_O·H_2_O, the Fe^III^ atom is coordinated by four N atoms of the  polybenzimidazole ligand and two Cl atoms in a distorted octa­hedral environment. The cation, anion, the uncoordinated water mol­ecule and the THF solvent molecule are linked by hydrogen bonds into a three-dimensional  network structure. The THF molecule is disordered with two sets of sites  in a 0.58 (1):0.42 (2) ratio..

## Related literature

For the synthesis of the ligand, see: Hendriks *et al.* (1982 ▶). For benzimidazole-like ligands, see: Moon & Soo Lah (2002 ▶). 
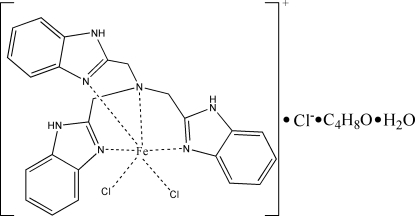


## Experimental

### 

#### Crystal data

[FeCl_2_(C_24_H_21_N_7_)]Cl·C_4_H_8_O·H_2_O
*M* *_r_* = 659.80
Monoclinic, 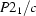

*a* = 10.2898 (4) Å
*b* = 13.7475 (5) Å
*c* = 21.5271 (7) Å
β = 101.614 (1)°
*V* = 2982.85 (19) Å^3^
*Z* = 4
Mo *K*α radiation
μ = 0.81 mm^−1^
*T* = 292 K
0.20 × 0.15 × 0.10 mm

#### Data collection

Bruker SMART APEX CCD area-detector diffractometer
Absorption correction: multi-scan (*SADABS*; Bruker, 2001 ▶) *T*_min_ = 0.844, *T*_max_ = 0.923
17337 measured reflections
5527 independent reflections
3999 reflections with *I* > 2σ(*I*)
*R*_int_ = 0.046

#### Refinement

*R*[*F* ^2^ > 2σ(*F* ^2^)] = 0.047
*wR*(*F* ^2^) = 0.116
*S* = 1.00
5527 reflections
404 parameters
21 restraints
H atoms treated by a mixture of independent and constrained refinement
Δρ_max_ = 0.33 e Å^−3^
Δρ_min_ = −0.25 e Å^−3^

### 

Data collection: *SMART* (Bruker, 2001 ▶); cell refinement: *SAINT-Plus* (Bruker, 2001 ▶); data reduction: *SAINT-Plus*; program(s) used to solve structure: *SHELXS97* (Sheldrick, 2008 ▶); program(s) used to refine structure: *SHELXL97* (Sheldrick, 2008 ▶); molecular graphics: *PLATON* (Spek, 2009 ▶); software used to prepare material for publication: *PLATON*.

## Supplementary Material

Crystal structure: contains datablocks global, I. DOI: 10.1107/S1600536809023423/ng2597sup1.cif

Structure factors: contains datablocks I. DOI: 10.1107/S1600536809023423/ng2597Isup2.hkl

Additional supplementary materials:  crystallographic information; 3D view; checkCIF report

## Figures and Tables

**Table 1:** Hydrogen-bond geometry (Å, °)

*D*—H⋯*A*	*D*—H	H⋯*A*	*D*⋯*A*	*D*—H⋯*A*
O1*W*—H1*A*⋯Cl3	0.828 (18)	2.34 (2)	3.136 (3)	160 (4)
O1*W*—H1*B*⋯Cl1	0.831 (19)	2.51 (3)	3.267 (3)	152 (4)
N2—H2⋯Cl3^i^	0.859 (18)	2.25 (2)	3.070 (3)	159 (3)
N4—H4⋯O1*W*^ii^	0.862 (18)	1.96 (2)	2.795 (4)	162 (3)
N6—H6⋯O1^iii^	0.852 (18)	1.91 (2)	2.741 (4)	163 (3)

Symmetry codes: (i) 
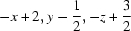
; (ii) 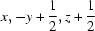; (iii) 
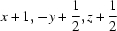
.

**Fig. 1.**
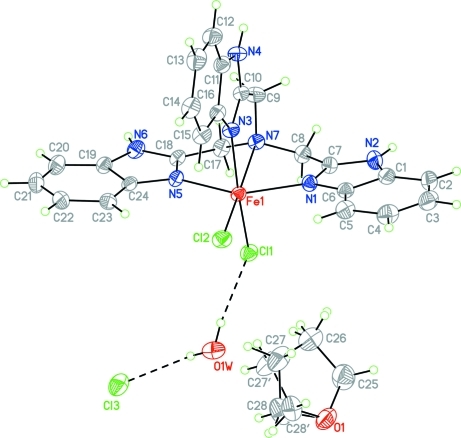
A view of the structure of (I), with displacement ellipsoids drawn at the 30% probability level. Hydrogen bonds are shown as dashed lines.

**Fig. 2.**
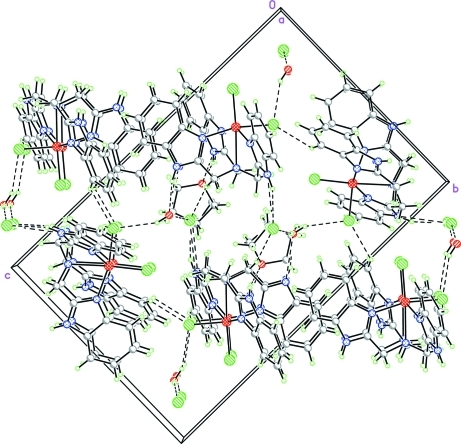
Plot of the crystal packing showing the linkage of the molecules by H-bonding shown as dashed lines.

## supplementary crystallographic information

### Comment

Tris(1-*H*-benzimidazol-2-ylmethl)amine-N, H_3_ntb, is a typical heterocyclic ligand with nitrogen as the donor atom. Over the past decades, there has been an interest in the Fe^III^ ions coordinated to benzimidazole-rich ligands in particular with respect to relationships between the activity and structural properties of metalloenzymes such as superoxide dismutases (SOD) (Moon & Soo Lah, 2002). Here, we report our findings in the title compound (I).

In compound (I) (Fig.1), the asymmetric unit consists of one [Fe(H_3_ntb).Cl_2_]^+^ cation, one Cl^-^ anion and each one water and tetrahydrofuran molecules. The Fe^III^ is coordinated by four nitrogen atoms of a tetradentate liangd ntb, and two chloride anions, forming a distorted octahedron. The Fe—N_amine_ bond length (2.355 (2) Å) is more longer than the mean bond length of Fe—N_benzimidazole_ (2.101 (2) Å), which is owing to the steric requirement. No other abnormal bond lengths and bond angles are observed in (I).

In the crystal, the ions are joined together by extensive hydrogen bondings and and π-π interactions, which lead to the formation of a three dimensional network (Fig.2).

### Experimental

All reagants and solvents were used as obtained without further purification. H_3_ntb was synthesized according to the literature (Hendriks *et al.*,1982). A mixture of FeCl_3_ (0.0324 g, 0.2 mmol), H_3_ntb(0.0814 g, 0.2 mmol), tetrahydrofuran (5 ml)was transferred to and sealed in a Parr Teflon-lined stainless steel vessel (15 ml), which was heated at 393 K for 3 days. After cooling to room temperature, the red block crystals of I were filtered off, washed with distilled water, and dried at ambient temperature (39 mg, yield 28.7% based on FeCl_3_).

### Refinement

During the refinement, C27 and C28 atoms were founded to be disordered over two positions and their occupancies were refined by using 'PART' command and some C–O and C–C distance of the THF molecule were refined by using command of 'DFIX'. The final satisfactory outcome was 0.58 (1):0.45 (2) for the major and minor components, respectively. H atoms bonded to carbon atoms were located at the geometrical positions with C—H=0.93Å (aromatic), 0.97 Å (methylene) and *U*_iso_(H) =1.2*U*_eq_(C) (aromatic and methlene). H atoms bonded N and O atoms were found from the difference maps with the constraints of N—H=0.86 (1) Å, O—H=0.82 (1) Å and H—H=1.35 (1) Å, with the thermal factors being set k times of their carrier atoms (k=1.2 for N and 1.5 for O atoms).

### Crystal data

**Table:**

[FeCl_2_(C_24_H_21_N_7_)]Cl·C_4_H_8_O·H_2_O	*F*(000) = 1364
*M**_r_* = 659.80	*D*_x_ = 1.469 Mg m^−^^3^
Monoclinic, *P*2_1_/*c*	Mo *K*α radiation, λ = 0.71073 Å
Hall symbol: -P 2ybc	Cell parameters from 3638 reflections
*a* = 10.2898 (4) Å	θ = 2.4–22.2°
*b* = 13.7475 (5) Å	µ = 0.81 mm^−^^1^
*c* = 21.5271 (7) Å	*T* = 292 K
β = 101.614 (1)°	Block, red
*V* = 2982.85 (19) Å^3^	0.20 × 0.15 × 0.10 mm
*Z* = 4	

### Data collection

**Table:**

Bruker SMART APEX CCD area-detector diffractometer	5527 independent reflections
Radiation source: fine focus sealed Siemens Mo tube	3999 reflections with *I* > 2σ(*I*)
graphite	*R*_int_ = 0.046
0.3° wide ω exposures scans	θ_max_ = 25.5°, θ_min_ = 2.4°
Absorption correction: multi-scan (*SADABS*; Sheldrick, 2001)	*h* = −12→12
*T*_min_ = 0.844, *T*_max_ = 0.923	*k* = −16→15
17337 measured reflections	*l* = −26→24

### Refinement

**Table:**

Refinement on *F*^2^	Primary atom site location: structure-invariant direct methods
Least-squares matrix: full	Secondary atom site location: difference Fourier map
*R*[*F*^2^ > 2σ(*F*^2^)] = 0.047	Hydrogen site location: inferred from neighbouring sites
*wR*(*F*^2^) = 0.116	H atoms treated by a mixture of independent and constrained refinement
*S* = 1.00	*w* = 1/[σ^2^(*F*_o_^2^) + (0.0587*P*)^2^] where *P* = (*F*_o_^2^ + 2*F*_c_^2^)/3
5527 reflections	(Δ/σ)_max_ < 0.001
404 parameters	Δρ_max_ = 0.33 e Å^−^^3^
21 restraints	Δρ_min_ = −0.25 e Å^−^^3^

### Special details

**Table:**

Geometry. All esds (except the esd in the dihedral angle between two l.s. planes) are estimated using the full covariance matrix. The cell esds are taken into account individually in the estimation of esds in distances, angles and torsion angles; correlations between esds in cell parameters are only used when they are defined by crystal symmetry. An approximate (isotropic) treatment of cell esds is used for estimating esds involving l.s. planes.
Refinement. Refinement of F^2^ against ALL reflections. The weighted R-factor wR and goodness of fit S are based on F^2^, conventional R-factors R are based on F, with F set to zero for negative F^2^. The threshold expression of F^2^ > 2sigma(F^2^) is used only for calculating R-factors(gt) etc. and is not relevant to the choice of reflections for refinement. R-factors based on F^2^ are statistically about twice as large as those based on F, and R- factors based on ALL data will be even larger.

### Fractional atomic coordinates and isotropic or equivalent isotropic displacement parameters (Å^2^)

**Table:**

	*x*	*y*	*z*	*U*_iso_*/*U*_eq_	Occ. (<1)
Fe1	0.94620 (4)	0.27332 (3)	0.810133 (19)	0.03211 (14)	
Cl1	1.00873 (8)	0.17478 (6)	0.73359 (4)	0.0445 (2)	
Cl2	0.86507 (8)	0.39490 (6)	0.74462 (4)	0.0476 (2)	
N1	0.7813 (2)	0.18448 (19)	0.81255 (12)	0.0369 (6)	
N2	0.6965 (3)	0.0441 (2)	0.83683 (14)	0.0482 (7)	
H2	0.694 (3)	−0.0126 (16)	0.8535 (15)	0.058*	
N3	0.9114 (2)	0.33689 (18)	0.89433 (11)	0.0367 (6)	
N4	0.9099 (3)	0.3256 (2)	0.99701 (12)	0.0439 (7)	
H4	0.924 (3)	0.296 (2)	1.0330 (11)	0.053*	
N5	1.1468 (2)	0.30502 (18)	0.84551 (11)	0.0342 (6)	
N6	1.3466 (2)	0.2668 (2)	0.89915 (13)	0.0446 (7)	
H6	1.413 (2)	0.237 (2)	0.9213 (14)	0.054*	
N7	1.0223 (2)	0.15132 (18)	0.88547 (11)	0.0361 (6)	
C1	0.5896 (3)	0.0998 (3)	0.80784 (15)	0.0452 (8)	
C2	0.4545 (3)	0.0799 (3)	0.79198 (18)	0.0604 (10)	
H2A	0.4191	0.0210	0.8019	0.072*	
C3	0.3760 (4)	0.1536 (3)	0.76056 (18)	0.0635 (11)	
H3	0.2851	0.1433	0.7487	0.076*	
C4	0.4273 (3)	0.2419 (3)	0.74607 (18)	0.0596 (10)	
H4A	0.3703	0.2893	0.7251	0.071*	
C5	0.5621 (3)	0.2610 (3)	0.76226 (16)	0.0508 (9)	
H5	0.5968	0.3203	0.7526	0.061*	
C6	0.6426 (3)	0.1889 (2)	0.79315 (14)	0.0392 (8)	
C7	0.8062 (3)	0.0962 (2)	0.83803 (15)	0.0407 (8)	
C8	0.9449 (3)	0.0627 (2)	0.86263 (16)	0.0428 (8)	
H8A	0.9477	0.0167	0.8970	0.051*	
H8B	0.9806	0.0315	0.8292	0.051*	
C9	0.9971 (3)	0.1834 (3)	0.94789 (15)	0.0483 (9)	
H9A	0.9372	0.1376	0.9619	0.058*	
H9B	1.0800	0.1825	0.9787	0.058*	
C10	0.9390 (3)	0.2821 (2)	0.94594 (14)	0.0368 (7)	
C11	0.8612 (3)	0.4177 (3)	0.97900 (15)	0.0424 (8)	
C12	0.8176 (3)	0.4915 (3)	1.01283 (17)	0.0539 (9)	
H12	0.8151	0.4852	1.0556	0.065*	
C13	0.7781 (3)	0.5753 (3)	0.9795 (2)	0.0586 (10)	
H13	0.7479	0.6271	1.0005	0.070*	
C14	0.7816 (3)	0.5853 (3)	0.91592 (18)	0.0516 (9)	
H14	0.7555	0.6438	0.8955	0.062*	
C15	0.8229 (3)	0.5102 (2)	0.88240 (16)	0.0451 (8)	
H15	0.8239	0.5166	0.8395	0.054*	
C16	0.8624 (3)	0.4259 (2)	0.91432 (15)	0.0378 (7)	
C17	1.1664 (3)	0.1394 (2)	0.88752 (16)	0.0412 (8)	
H17A	1.2099	0.1126	0.9281	0.049*	
H17B	1.1807	0.0956	0.8542	0.049*	
C18	1.2211 (3)	0.2369 (2)	0.87831 (14)	0.0349 (7)	
C19	1.3560 (3)	0.3620 (2)	0.87884 (14)	0.0402 (8)	
C20	1.4596 (3)	0.4284 (3)	0.88702 (17)	0.0552 (10)	
H20	1.5444	0.4124	0.9089	0.066*	
C21	1.4307 (3)	0.5183 (3)	0.86141 (17)	0.0568 (10)	
H21	1.4979	0.5645	0.8657	0.068*	
C22	1.3051 (3)	0.5433 (3)	0.82922 (16)	0.0514 (9)	
H22	1.2898	0.6062	0.8136	0.062*	
C23	1.2022 (3)	0.4777 (2)	0.81965 (15)	0.0421 (8)	
H23	1.1181	0.4944	0.7973	0.051*	
C24	1.2294 (3)	0.3859 (2)	0.84485 (14)	0.0361 (7)	
O1W	0.9939 (3)	0.2955 (2)	0.60165 (12)	0.0715 (8)	
H1A	1.073 (2)	0.302 (4)	0.599 (2)	0.107*	
H1B	0.984 (4)	0.283 (4)	0.6383 (12)	0.107*	
Cl3	1.26515 (10)	0.36822 (8)	0.57345 (5)	0.0690 (3)	
O1	0.5693 (3)	0.2940 (2)	0.48243 (13)	0.0781 (9)	
C25	0.5735 (5)	0.2366 (3)	0.5376 (2)	0.0854 (14)	
H25A	0.6237	0.1776	0.5349	0.102*	
H25B	0.4842	0.2184	0.5413	0.102*	
C26	0.6369 (5)	0.2936 (4)	0.5937 (2)	0.0999 (17)	
H26A	0.7268	0.2714	0.6098	0.120*	0.58
H26B	0.5867	0.2888	0.6272	0.120*	0.58
H26C	0.7138	0.2598	0.6174	0.120*	0.42
H26D	0.5754	0.3058	0.6215	0.120*	0.42
C27	0.6360 (17)	0.3975 (6)	0.5690 (5)	0.088 (6)	0.58
H27A	0.7114	0.4345	0.5913	0.106*	0.58
H27B	0.5545	0.4312	0.5717	0.106*	0.58
C28	0.6462 (10)	0.3772 (7)	0.4990 (5)	0.084 (4)	0.58
H28A	0.6115	0.4314	0.4719	0.101*	0.58
H28B	0.7376	0.3657	0.4956	0.101*	0.58
C28'	0.5865 (15)	0.3944 (7)	0.4996 (6)	0.085 (5)	0.42
H28C	0.5024	0.4247	0.5018	0.102*	0.42
H28D	0.6277	0.4299	0.4698	0.102*	0.42
C27'	0.679 (2)	0.3894 (9)	0.5661 (6)	0.084 (7)	0.42
H27C	0.7717	0.3873	0.5626	0.101*	0.42
H27D	0.6653	0.4447	0.5920	0.101*	0.42

### Atomic displacement parameters (Å^2^)

**Table:**

	*U*^11^	*U*^22^	*U*^33^	*U*^12^	*U*^13^	*U*^23^
Fe1	0.0333 (2)	0.0318 (3)	0.0293 (2)	0.00082 (18)	0.00167 (17)	0.00084 (19)
Cl1	0.0463 (4)	0.0451 (5)	0.0424 (5)	0.0001 (4)	0.0096 (3)	−0.0090 (4)
Cl2	0.0550 (5)	0.0437 (5)	0.0397 (5)	0.0064 (4)	−0.0010 (4)	0.0092 (4)
N1	0.0365 (14)	0.0339 (16)	0.0391 (15)	−0.0016 (11)	0.0046 (11)	0.0021 (12)
N2	0.0503 (17)	0.0416 (18)	0.0534 (19)	−0.0107 (15)	0.0116 (14)	0.0037 (15)
N3	0.0415 (14)	0.0363 (16)	0.0308 (14)	0.0033 (11)	0.0037 (11)	−0.0003 (12)
N4	0.0504 (16)	0.052 (2)	0.0296 (15)	0.0020 (14)	0.0084 (13)	0.0017 (14)
N5	0.0340 (13)	0.0291 (14)	0.0363 (15)	−0.0007 (11)	−0.0006 (11)	−0.0004 (12)
N6	0.0359 (15)	0.0472 (19)	0.0453 (17)	0.0058 (13)	−0.0050 (12)	0.0021 (14)
N7	0.0405 (14)	0.0316 (15)	0.0348 (14)	0.0048 (11)	0.0041 (11)	0.0013 (12)
C1	0.0414 (18)	0.055 (2)	0.0401 (19)	−0.0032 (16)	0.0109 (15)	−0.0033 (17)
C2	0.049 (2)	0.078 (3)	0.058 (2)	−0.020 (2)	0.0176 (18)	−0.010 (2)
C3	0.0372 (19)	0.098 (3)	0.056 (2)	−0.004 (2)	0.0101 (17)	−0.018 (2)
C4	0.042 (2)	0.077 (3)	0.057 (2)	0.0144 (19)	0.0026 (17)	0.000 (2)
C5	0.049 (2)	0.054 (2)	0.050 (2)	0.0040 (17)	0.0093 (17)	−0.0025 (18)
C6	0.0365 (17)	0.047 (2)	0.0348 (18)	0.0014 (15)	0.0079 (14)	−0.0036 (15)
C7	0.0407 (17)	0.040 (2)	0.0416 (19)	−0.0035 (15)	0.0084 (14)	−0.0015 (16)
C8	0.0458 (18)	0.0325 (19)	0.048 (2)	0.0013 (14)	0.0037 (15)	0.0033 (16)
C9	0.061 (2)	0.047 (2)	0.0361 (19)	0.0098 (17)	0.0095 (16)	0.0042 (16)
C10	0.0384 (16)	0.040 (2)	0.0309 (17)	−0.0004 (14)	0.0047 (13)	0.0000 (15)
C11	0.0401 (17)	0.044 (2)	0.043 (2)	−0.0008 (15)	0.0082 (15)	−0.0065 (16)
C12	0.057 (2)	0.058 (3)	0.050 (2)	−0.0008 (18)	0.0179 (17)	−0.016 (2)
C13	0.056 (2)	0.049 (2)	0.074 (3)	0.0028 (18)	0.0196 (19)	−0.023 (2)
C14	0.0475 (19)	0.043 (2)	0.062 (2)	0.0049 (16)	0.0045 (17)	−0.0037 (19)
C15	0.0487 (19)	0.037 (2)	0.048 (2)	0.0072 (15)	0.0054 (15)	−0.0022 (17)
C16	0.0340 (16)	0.040 (2)	0.0391 (19)	0.0007 (14)	0.0059 (13)	−0.0056 (15)
C17	0.0386 (17)	0.0341 (19)	0.048 (2)	0.0068 (14)	0.0005 (14)	0.0033 (16)
C18	0.0360 (16)	0.0300 (18)	0.0352 (17)	0.0039 (13)	−0.0010 (13)	−0.0027 (14)
C19	0.0375 (17)	0.041 (2)	0.0401 (19)	−0.0015 (14)	0.0025 (14)	−0.0003 (16)
C20	0.0357 (18)	0.070 (3)	0.054 (2)	−0.0071 (17)	−0.0051 (16)	0.000 (2)
C21	0.051 (2)	0.056 (3)	0.061 (2)	−0.0186 (18)	0.0048 (18)	0.004 (2)
C22	0.058 (2)	0.047 (2)	0.048 (2)	−0.0103 (17)	0.0088 (17)	0.0069 (18)
C23	0.0450 (18)	0.042 (2)	0.0383 (18)	−0.0008 (15)	0.0056 (14)	0.0053 (16)
C24	0.0380 (16)	0.039 (2)	0.0295 (16)	−0.0019 (14)	0.0038 (13)	−0.0051 (14)
O1W	0.082 (2)	0.088 (2)	0.0449 (16)	−0.0061 (18)	0.0147 (14)	−0.0079 (16)
Cl3	0.0691 (6)	0.0644 (7)	0.0706 (7)	−0.0083 (5)	0.0075 (5)	−0.0187 (5)
O1	0.081 (2)	0.082 (2)	0.0597 (19)	−0.0229 (17)	−0.0139 (14)	−0.0003 (17)
C25	0.083 (3)	0.075 (3)	0.093 (4)	−0.005 (2)	0.006 (3)	0.013 (3)
C26	0.102 (4)	0.119 (5)	0.072 (3)	−0.026 (3)	0.002 (3)	−0.006 (3)
C27	0.083 (9)	0.089 (10)	0.086 (10)	−0.010 (7)	0.001 (6)	−0.027 (7)
C28	0.072 (7)	0.078 (7)	0.105 (9)	−0.002 (5)	0.024 (6)	0.026 (6)
C28'	0.088 (12)	0.079 (11)	0.091 (11)	−0.024 (8)	0.023 (9)	0.000 (8)
C27'	0.087 (14)	0.069 (11)	0.080 (13)	0.020 (7)	−0.021 (8)	−0.021 (8)

### Geometric parameters (Å, °)

**Table:**

Fe1—N5	2.097 (2)	C13—C14	1.384 (5)
Fe1—N1	2.100 (2)	C13—H13	0.9300
Fe1—N3	2.107 (2)	C14—C15	1.375 (4)
Fe1—Cl2	2.2373 (9)	C14—H14	0.9300
Fe1—Cl1	2.3219 (9)	C15—C16	1.367 (4)
Fe1—N7	2.355 (2)	C15—H15	0.9300
Cl1—H1B	2.51 (3)	C17—C18	1.482 (4)
N1—C7	1.335 (4)	C17—H17A	0.9700
N1—C6	1.405 (4)	C17—H17B	0.9700
N2—C7	1.333 (4)	C19—C20	1.387 (4)
N2—C1	1.382 (4)	C19—C24	1.399 (4)
N2—H2	0.859 (18)	C20—C21	1.361 (5)
N3—C10	1.325 (4)	C20—H20	0.9300
N3—C16	1.422 (4)	C21—C22	1.381 (5)
N4—C10	1.338 (4)	C21—H21	0.9300
N4—C11	1.388 (4)	C22—C23	1.375 (4)
N4—H4	0.862 (18)	C22—H22	0.9300
N5—C18	1.320 (4)	C23—C24	1.379 (4)
N5—C24	1.402 (4)	C23—H23	0.9300
N6—C18	1.343 (4)	O1W—H1A	0.828 (18)
N6—C19	1.389 (4)	O1W—H1B	0.831 (19)
N6—H6	0.852 (18)	Cl3—H1A	2.34 (2)
N7—C8	1.484 (4)	O1—C28	1.395 (8)
N7—C17	1.484 (4)	O1—C25	1.420 (5)
N7—C9	1.486 (4)	O1—C28'	1.430 (9)
C1—C2	1.391 (4)	C25—C26	1.476 (5)
C1—C6	1.403 (4)	C25—H25A	0.9700
C2—C3	1.384 (5)	C25—H25B	0.9700
C2—H2A	0.9300	C26—C27	1.523 (8)
C3—C4	1.385 (6)	C26—C27'	1.544 (9)
C3—H3	0.9300	C26—H26A	0.9700
C4—C5	1.386 (5)	C26—H26B	0.9700
C4—H4A	0.9300	C26—H26C	0.9700
C5—C6	1.374 (5)	C26—H26D	0.9700
C5—H5	0.9300	C27—C28	1.556 (9)
C7—C8	1.492 (4)	C27—H27A	0.9700
C8—H8A	0.9700	C27—H27B	0.9700
C8—H8B	0.9700	C28—H28A	0.9700
C9—C10	1.479 (4)	C28—H28B	0.9700
C9—H9A	0.9700	C28'—C27'	1.553 (10)
C9—H9B	0.9700	C28'—H28C	0.9700
C11—C12	1.376 (5)	C28'—H28D	0.9700
C11—C16	1.400 (4)	C27'—H27C	0.9700
C12—C13	1.374 (5)	C27'—H27D	0.9700
C12—H12	0.9300		
			
N5—Fe1—N1	148.56 (10)	C15—C14—H14	119.4
N5—Fe1—N3	85.80 (9)	C13—C14—H14	119.4
N1—Fe1—N3	86.65 (10)	C16—C15—C14	117.7 (3)
N5—Fe1—Cl2	106.78 (7)	C16—C15—H15	121.1
N1—Fe1—Cl2	104.43 (7)	C14—C15—H15	121.1
N3—Fe1—Cl2	97.23 (7)	C15—C16—C11	120.4 (3)
N5—Fe1—Cl1	89.19 (7)	C15—C16—N3	131.7 (3)
N1—Fe1—Cl1	91.09 (7)	C11—C16—N3	107.9 (3)
N3—Fe1—Cl1	166.40 (7)	C18—C17—N7	107.5 (2)
Cl2—Fe1—Cl1	96.32 (3)	C18—C17—H17A	110.2
N5—Fe1—N7	74.41 (9)	N7—C17—H17A	110.2
N1—Fe1—N7	74.16 (9)	C18—C17—H17B	110.2
N3—Fe1—N7	78.09 (9)	N7—C17—H17B	110.2
Cl2—Fe1—N7	175.13 (7)	H17A—C17—H17B	108.5
Cl1—Fe1—N7	88.40 (6)	N5—C18—N6	112.1 (3)
Fe1—Cl1—H1B	103.7 (10)	N5—C18—C17	121.2 (3)
C7—N1—C6	105.1 (2)	N6—C18—C17	126.7 (3)
C7—N1—Fe1	116.72 (19)	C20—C19—N6	132.9 (3)
C6—N1—Fe1	138.1 (2)	C20—C19—C24	121.5 (3)
C7—N2—C1	107.7 (3)	N6—C19—C24	105.6 (3)
C7—N2—H2	125 (2)	C21—C20—C19	116.5 (3)
C1—N2—H2	127 (2)	C21—C20—H20	121.8
C10—N3—C16	105.4 (2)	C19—C20—H20	121.8
C10—N3—Fe1	116.1 (2)	C20—C21—C22	122.4 (3)
C16—N3—Fe1	138.5 (2)	C20—C21—H21	118.8
C10—N4—C11	107.9 (3)	C22—C21—H21	118.8
C10—N4—H4	120 (2)	C23—C22—C21	121.8 (3)
C11—N4—H4	132 (2)	C23—C22—H22	119.1
C18—N5—C24	106.3 (2)	C21—C22—H22	119.1
C18—N5—Fe1	118.0 (2)	C22—C23—C24	116.8 (3)
C24—N5—Fe1	135.7 (2)	C22—C23—H23	121.6
C18—N6—C19	107.9 (2)	C24—C23—H23	121.6
C18—N6—H6	130 (2)	C23—C24—C19	121.0 (3)
C19—N6—H6	122 (2)	C23—C24—N5	130.9 (3)
C8—N7—C17	112.7 (2)	C19—C24—N5	108.1 (3)
C8—N7—C9	111.3 (2)	H1A—O1W—H1B	113 (3)
C17—N7—C9	111.2 (2)	C28—O1—C25	108.8 (5)
C8—N7—Fe1	105.93 (17)	C25—O1—C28'	109.8 (6)
C17—N7—Fe1	106.90 (17)	C28—O1—H4A	93.5
C9—N7—Fe1	108.49 (18)	O1—C25—C26	108.8 (3)
N2—C1—C2	131.9 (3)	O1—C25—H25A	109.9
N2—C1—C6	106.0 (3)	C26—C25—H25A	109.9
C2—C1—C6	122.0 (3)	O1—C25—H25B	109.9
C3—C2—C1	115.6 (4)	C26—C25—H25B	109.9
C3—C2—H2A	122.2	H25A—C25—H25B	108.3
C1—C2—H2A	122.2	C25—C26—C27	104.0 (5)
C2—C3—C4	122.7 (3)	C25—C26—C27'	104.5 (6)
C2—C3—H3	118.6	C25—C26—H26A	111.0
C4—C3—H3	118.6	C27—C26—H26A	111.0
C3—C4—C5	121.1 (4)	C27'—C26—H26A	94.8
C3—C4—H4A	119.4	C25—C26—H26B	111.0
C5—C4—H4A	119.4	C27—C26—H26B	111.0
C6—C5—C4	117.4 (4)	C27'—C26—H26B	125.4
C6—C5—H5	121.3	H26A—C26—H26B	109.0
C4—C5—H5	121.3	C25—C26—H26C	110.8
C5—C6—C1	121.1 (3)	C27—C26—H26C	125.4
C5—C6—N1	130.7 (3)	C27'—C26—H26C	110.4
C1—C6—N1	108.2 (3)	H26B—C26—H26C	94.3
N2—C7—N1	112.9 (3)	C25—C26—H26D	111.2
N2—C7—C8	126.0 (3)	C27'—C26—H26D	111.1
N1—C7—C8	121.1 (3)	H26A—C26—H26D	121.8
N7—C8—C7	106.0 (2)	H26C—C26—H26D	108.7
N7—C8—H8A	110.5	C26—C27—C28	100.0 (7)
C7—C8—H8A	110.5	C26—C27—H27A	111.8
N7—C8—H8B	110.5	C28—C27—H27A	111.8
C7—C8—H8B	110.5	C26—C27—H27B	111.8
H8A—C8—H8B	108.7	C28—C27—H27B	111.8
C10—C9—N7	112.9 (3)	H27A—C27—H27B	109.5
C10—C9—H9A	109.0	O1—C28—C27	104.4 (7)
N7—C9—H9A	109.0	O1—C28—H28A	110.9
C10—C9—H9B	109.0	C27—C28—H28A	110.9
N7—C9—H9B	109.0	O1—C28—H28B	110.9
H9A—C9—H9B	107.8	C27—C28—H28B	110.9
N3—C10—N4	112.9 (3)	H28A—C28—H28B	108.9
N3—C10—C9	124.3 (3)	O1—C28'—C27'	102.5 (8)
N4—C10—C9	122.9 (3)	O1—C28'—H28C	111.3
C12—C11—N4	131.5 (3)	C27'—C28'—H28C	111.3
C12—C11—C16	122.5 (3)	O1—C28'—H28D	111.3
N4—C11—C16	106.0 (3)	C27'—C28'—H28D	111.3
C13—C12—C11	115.9 (3)	H28C—C28'—H28D	109.2
C13—C12—H12	122.1	C26—C27'—C28'	102.9 (8)
C11—C12—H12	122.1	C26—C27'—H27C	111.2
C12—C13—C14	122.4 (3)	C28'—C27'—H27C	111.2
C12—C13—H13	118.8	C26—C27'—H27D	111.2
C14—C13—H13	118.8	C28'—C27'—H27D	111.2
C15—C14—C13	121.1 (4)	H27C—C27'—H27D	109.1
			
N5—Fe1—Cl1—H1B	−96.2 (10)	N1—C7—C8—N7	30.8 (4)
N1—Fe1—Cl1—H1B	115.2 (10)	C8—N7—C9—C10	−118.9 (3)
N3—Fe1—Cl1—H1B	−164.5 (10)	C17—N7—C9—C10	114.5 (3)
Cl2—Fe1—Cl1—H1B	10.6 (10)	Fe1—N7—C9—C10	−2.7 (3)
N7—Fe1—Cl1—H1B	−170.7 (10)	C16—N3—C10—N4	1.5 (3)
N5—Fe1—N1—C7	−20.2 (3)	Fe1—N3—C10—N4	−177.65 (19)
N3—Fe1—N1—C7	−96.6 (2)	C16—N3—C10—C9	−177.9 (3)
Cl2—Fe1—N1—C7	166.8 (2)	Fe1—N3—C10—C9	2.9 (4)
Cl1—Fe1—N1—C7	70.0 (2)	C11—N4—C10—N3	−1.2 (3)
N7—Fe1—N1—C7	−18.0 (2)	C11—N4—C10—C9	178.3 (3)
N5—Fe1—N1—C6	161.4 (3)	N7—C9—C10—N3	0.2 (5)
N3—Fe1—N1—C6	85.1 (3)	N7—C9—C10—N4	−179.2 (3)
Cl2—Fe1—N1—C6	−11.6 (3)	C10—N4—C11—C12	−179.8 (3)
Cl1—Fe1—N1—C6	−108.4 (3)	C10—N4—C11—C16	0.4 (3)
N7—Fe1—N1—C6	163.6 (3)	N4—C11—C12—C13	178.6 (3)
N5—Fe1—N3—C10	−78.2 (2)	C16—C11—C12—C13	−1.5 (5)
N1—Fe1—N3—C10	71.3 (2)	C11—C12—C13—C14	0.0 (5)
Cl2—Fe1—N3—C10	175.4 (2)	C12—C13—C14—C15	1.3 (5)
Cl1—Fe1—N3—C10	−9.5 (4)	C13—C14—C15—C16	−1.0 (5)
N7—Fe1—N3—C10	−3.2 (2)	C14—C15—C16—C11	−0.5 (5)
N5—Fe1—N3—C16	103.0 (3)	C14—C15—C16—N3	−179.0 (3)
N1—Fe1—N3—C16	−107.5 (3)	C12—C11—C16—C15	1.8 (5)
Cl2—Fe1—N3—C16	−3.4 (3)	N4—C11—C16—C15	−178.3 (3)
Cl1—Fe1—N3—C16	171.7 (2)	C12—C11—C16—N3	−179.3 (3)
N7—Fe1—N3—C16	178.0 (3)	N4—C11—C16—N3	0.5 (3)
N1—Fe1—N5—C18	16.6 (3)	C10—N3—C16—C15	177.4 (3)
N3—Fe1—N5—C18	93.2 (2)	Fe1—N3—C16—C15	−3.7 (5)
Cl2—Fe1—N5—C18	−170.5 (2)	C10—N3—C16—C11	−1.2 (3)
Cl1—Fe1—N5—C18	−74.2 (2)	Fe1—N3—C16—C11	177.6 (2)
N7—Fe1—N5—C18	14.4 (2)	C8—N7—C17—C18	151.7 (3)
N1—Fe1—N5—C24	−161.7 (2)	C9—N7—C17—C18	−82.5 (3)
N3—Fe1—N5—C24	−85.1 (3)	Fe1—N7—C17—C18	35.7 (3)
Cl2—Fe1—N5—C24	11.2 (3)	C24—N5—C18—N6	−0.2 (3)
Cl1—Fe1—N5—C24	107.6 (3)	Fe1—N5—C18—N6	−178.9 (2)
N7—Fe1—N5—C24	−163.9 (3)	C24—N5—C18—C17	−178.6 (3)
N5—Fe1—N7—C8	−148.3 (2)	Fe1—N5—C18—C17	2.7 (4)
N1—Fe1—N7—C8	32.90 (18)	C19—N6—C18—N5	0.2 (4)
N3—Fe1—N7—C8	122.78 (19)	C19—N6—C18—C17	178.5 (3)
Cl1—Fe1—N7—C8	−58.69 (18)	N7—C17—C18—N5	−28.4 (4)
N5—Fe1—N7—C17	−27.88 (18)	N7—C17—C18—N6	153.5 (3)
N1—Fe1—N7—C17	153.3 (2)	C18—N6—C19—C20	179.1 (4)
N3—Fe1—N7—C17	−116.80 (19)	C18—N6—C19—C24	−0.2 (3)
Cl1—Fe1—N7—C17	61.73 (17)	N6—C19—C20—C21	−177.9 (4)
N5—Fe1—N7—C9	92.1 (2)	C24—C19—C20—C21	1.3 (5)
N1—Fe1—N7—C9	−86.7 (2)	C19—C20—C21—C22	0.4 (6)
N3—Fe1—N7—C9	3.18 (19)	C20—C21—C22—C23	−1.7 (6)
Cl1—Fe1—N7—C9	−178.29 (19)	C21—C22—C23—C24	1.2 (5)
C7—N2—C1—C2	176.9 (3)	C22—C23—C24—C19	0.5 (5)
C7—N2—C1—C6	−1.2 (4)	C22—C23—C24—N5	177.4 (3)
N2—C1—C2—C3	−177.6 (3)	C20—C19—C24—C23	−1.8 (5)
C6—C1—C2—C3	0.3 (5)	N6—C19—C24—C23	177.6 (3)
C1—C2—C3—C4	−0.6 (5)	C20—C19—C24—N5	−179.3 (3)
C2—C3—C4—C5	0.4 (6)	N6—C19—C24—N5	0.1 (3)
C3—C4—C5—C6	0.0 (5)	C18—N5—C24—C23	−177.2 (3)
C4—C5—C6—C1	−0.3 (5)	Fe1—N5—C24—C23	1.3 (5)
C4—C5—C6—N1	176.4 (3)	C18—N5—C24—C19	0.0 (3)
N2—C1—C6—C5	178.5 (3)	Fe1—N5—C24—C19	178.5 (2)
C2—C1—C6—C5	0.1 (5)	C28—O1—C25—C26	10.0 (7)
N2—C1—C6—N1	1.2 (3)	C28'—O1—C25—C26	−18.6 (8)
C2—C1—C6—N1	−177.2 (3)	H4A—O1—C25—C26	−61.4
C7—N1—C6—C5	−177.6 (3)	O1—C25—C26—C27	14.7 (8)
Fe1—N1—C6—C5	0.9 (5)	O1—C25—C26—C27'	−3.7 (11)
C7—N1—C6—C1	−0.6 (3)	C25—C26—C27—C28	−30.5 (10)
Fe1—N1—C6—C1	177.9 (2)	C27'—C26—C27—C28	64 (2)
C1—N2—C7—N1	0.9 (4)	C25—O1—C28—C27	−30.0 (10)
C1—N2—C7—C8	−177.3 (3)	C28'—O1—C28—C27	67.0 (18)
C6—N1—C7—N2	−0.1 (4)	H4A—O1—C28—C27	6.6
Fe1—N1—C7—N2	−179.0 (2)	C26—C27—C28—O1	37.2 (11)
C6—N1—C7—C8	178.1 (3)	C28—O1—C28'—C27'	−60.6 (18)
Fe1—N1—C7—C8	−0.8 (4)	C25—O1—C28'—C27'	32.1 (13)
C17—N7—C8—C7	−157.2 (2)	H4A—O1—C28'—C27'	57.8
C9—N7—C8—C7	77.1 (3)	C25—C26—C27'—C28'	22.1 (15)
Fe1—N7—C8—C7	−40.6 (3)	C27—C26—C27'—C28'	−68 (2)
N2—C7—C8—N7	−151.2 (3)	O1—C28'—C27'—C26	−32.6 (16)

### Hydrogen-bond geometry (Å, °)

**Table:**

*D*—H···*A*	*D*—H	H···*A*	*D*···*A*	*D*—H···*A*
O1W—H1A···Cl3	0.83 (2)	2.34 (2)	3.136 (3)	160 (4)
O1W—H1B···Cl1	0.83 (2)	2.51 (3)	3.267 (3)	152 (4)
C23—H23···Cl1^i^	0.93	2.82	3.516 (3)	133
N2—H2···Cl3^ii^	0.86 (2)	2.25 (2)	3.070 (3)	159 (3)
N4—H4···O1W^iii^	0.86 (2)	1.96 (2)	2.795 (4)	162 (3)
N6—H6···O1^iv^	0.85 (2)	1.91 (2)	2.741 (4)	163 (3)
C9—H9B···Cl3^iii^	0.97	2.59	3.523 (3)	162
C3—H3···Cl1^v^	0.93	2.83	3.718 (4)	160
C15—H15···Cl2	0.93	2.74	3.466 (3)	136

Symmetry codes: (i) −*x*+2, *y*+1/2, −*z*+3/2; (ii) −*x*+2, *y*−1/2, −*z*+3/2; (iii) *x*, −*y*+1/2, *z*+1/2; (iv) *x*+1, −*y*+1/2, *z*+1/2; (v) *x*−1, *y*, *z*.
